# Emollient use alters skin barrier and microbes in infants at risk for developing atopic dermatitis

**DOI:** 10.1371/journal.pone.0192443

**Published:** 2018-02-28

**Authors:** Martin Glatz, Jay-Hyun Jo, Elizabeth A. Kennedy, Eric C. Polley, Julia A. Segre, Eric L. Simpson, Heidi H. Kong

**Affiliations:** 1 Dermatology Branch, Center for Cancer Research, National Cancer Institute, NIH, Bethesda, Maryland, United States of America; 2 Biometric Research Branch, Division of Cancer Treatment and Diagnosis, National Cancer Institute, NIH, Rockville, Maryland, United States of America; 3 Translational and Functional Genomics Branch, NHGRI, Bethesda, Maryland, United States of America; 4 Department of Dermatology, Oregon Health & Science University, Portland, Oregon, United States of America; University of Colorado, Boulder, UNITED STATES

## Abstract

**Background:**

Emollients are a mainstay of treatment in atopic dermatitis (AD), a disease distinguished by skin bacterial dysbiosis. However, changes in skin microbiota when emollients are used as a potential AD preventative measure in infants remain incompletely characterized.

**Results:**

We compared skin barrier parameters, AD development, and bacterial 16S ribosomal RNA gene sequences of cheek, dorsal and volar forearm samples from 6-month-old infants with a family history of atopy randomized to receive emollients (n = 11) or no emollients (controls, n = 12). The emollient group had a lower skin pH than the control group. The number of bacterial taxa in the emollient group was higher than in the control group at all sites. The *Streptococcus salivarius* proportion was higher in the emollient versus control groups at all sites. *S*. *salivarius* proportion appeared higher in infants without AD compared to infants with AD. A decrease in *S*. *salivarius* abundance was further identified in a separate larger population of older children demonstrating an inverse correlation between AD severity at sampling sites and *S*. *salivarius* proportions.

**Conclusions:**

The decreased skin pH and the increased proportion of *S*. *salivarius* after long-term emollient use in infants at risk for developing AD may contribute to the preventative effects of emollients in high-risk infants.

## Introduction

Atopic dermatitis (AD) is a chronic relapsing inflammatory pruritic skin disorder, which develops in the first six months of life in 45% of patients [1, 2]. Infants with a positive family history of atopy, i.e. first-degree relatives with AD, asthma, or allergic rhinitis, have an increased risk of developing AD with an odds ratio of 2–2.7 as compared to infants without a family history of atopy [3–5].

AD is a multifactorial disease, potentially encompassing different disease phenotypes under a single diagnosis. AD is characterized by impaired skin barrier function [1, 6], which may precede clinical skin lesions in infants [7, 8]. The impaired skin barrier function in AD promotes altered microbial colonization of atopic skin [9, 10]. Cultivation studies have shown that *Staphylococcus aureus* colonizes the lesional and non-lesional skin of AD patients in 91–100% and 31–78%, respectively, but the skin of healthy individuals in only 10% [11, 12]. The significance of altered bacterial colonization for AD is demonstrated by the positive correlation between AD severity and density of cutaneous *S*. *aureus* colonization [13]. However, while a high proportion of AD patients are culture-positive for *S*. *aureus*, some AD patients are not culture-positive *S*. *aureus*, underscoring disease heterogeneity. High-throughput 16S rRNA gene sequencing enables examination of many genera and species including rare and fastidious bacteria, and therefore provides a higher resolution examination of skin bacterial communities [14–16]. 16S rRNA gene sequencing of AD skin samples has not only underscored culture-based results of increased *S*. *aureus* during AD flares, but has also demonstrated significant shifts of *S*. *epidermidis* and more fastidious or rare bacteria, i.e. *Corynebacterium* spp., *Propionibacterium* spp., *Acinetobacter* spp. or *Gemella* spp., during disease flares and after treatment [15].

Skin emollients are a critical component of AD treatment because they rehydrate the skin, improve the skin barrier function [17–19], and reduce the severity of AD [18, 20]. Furthermore, emollients may prevent the development of eczema in infants at high risk for AD when used regularly in the first six months of life [21, 22]. In addition to improving the skin barrier, the effects of the emollients and their components may alter skin bacterial growth and survival. Despite the frequent application of emollients to atopic skin and the significance of bacterial colonization of skin for the course of AD, studies investigating the effects of emollients on skin bacterial communities are few with mixed results. For example, studies from Japan and the Philippines demonstrated that the detection rate of *S*. *aureus* on the lesions of infants and adults with AD was reduced by 50–78% after a twice-daily application of emollients, including ointments, coconut oil, or olive oil for 1–4 months [23, 24]. Accordingly, use of emollients in preterm neonates reduced the risk for skin infections [25]. In contrast, an observational study of children with AD in New Zealand showed no association between emollient use and *S*. *aureus* culture positivity; however, the use of emollients, topical antimicrobials, and topical anti-inflammatory medications were not controlled in the observational study [26]. Also, water-in-oil emulsion use in non-atopic premature neonates [27] did not affect rates of positive bacterial cultures on abdomen and axilla. Since prior studies were based on cultivation methods, used various forms of emollients [23, 24], allowed use of concomitant topical medications, or were potentially confounded by effects of active AD lesions on skin microbial communities, we sought to investigate the effects of a single type of emollient on skin barrier features and bacterial communities by high-throughput 16S rRNA gene sequencing in an infant population at risk for developing AD.

## Methods

### Subjects and study design

Participating infants were the enrolled subjects from one of the four centers that participated in a larger trial designed to assess the safety, adherence and acceptability of an emollient treatment study in infants [21]. The trial was approved by the Institutional Review Board of Oregon Health & Science University and registered with clinicaltrials.gov (NCT01142999A) and Current Controlled Trials ISRCTN84854178. All infants for the present study were from the Oregon Health & Science University in Portland, Oregon site. Guardians of all infants gave written informed consent to participate upon enrollment. We included newborns considered to be at risk for developing AD, i.e. parent or sibling with a history of AD, asthma or allergic rhinitis. Exclusion criteria at enrollment included preterm birth (prior to 37 weeks gestation), major congenital anomaly, hydrops fetalis, or significant dermatitis at birth. Exclusion criteria included receiving systemic or topical antibiotics during the study. Newborns were randomized within 3 weeks of birth to receive either the once daily application of emollient (Cetaphil Moisturizing Cream^™^, Galderma Laboratories, Fort Worth, TX) as previously studied [28] or no routine emollient use (randomization groups). Emollient application was started within 3 weeks of birth to all body surfaces except the scalp and diaper area for 6 months. Both randomization groups received the same general advice regarding proper bathing techniques and avoiding soap. Research personnel contacted parents by telephone at 10 days, 6 weeks, and 18 weeks to discuss the subject’s skin and record use of topical emollients. Subjects were also evaluated at clinic visits at 12 and 24 weeks, including a blinded assessment of the skin. In addition to the scheduled clinic visits, parents were asked to contact the research team if there were concerns about the subject’s skin. If parents reported symptoms of eczema, then an additional visit with the dermatologist was arranged for evaluation of the presence of eczematous lesions in typical locations for infants consistent with atopic dermatitis.

### Sample collection and processing

Prior to sample collection, parents/guardians of subjects were asked to avoid applying any emollients and to avoid bathing for at least 24 hours. At 6 months of age, samples were collected by one individual, based on a previously published standardized protocol [16, 29]. Briefly, skin samples were obtained rubbing a flocked swab (Catch-All^™^ Sample Collection Swab, Epicentre, Madison, WI) pre-moistened with yeast cell lysis buffer (MPY80200, Epicenter, Madison, WI) on the cheek, dorsal forearm and volar forearm. Controls to monitor for contamination were obtained at each sampling session by pre-moistening swabs and exposing them to air without skin contact. Swabs were stored in lysis buffer at -80°C following collection. For extractions, skin and control swabs were incubated in Yeast Cell Lysis buffer and ReadyLyse Lysozyme Solution (Epicentre) for 1 hour with shaking at 37°C. 5-mm steel beads were added to mechanically disrupt cell walls using a Tissuelyser (Qiagen) for 2 minutes at 30 Hz, followed by 30 minute incubation at 65°C for complete lysis. MPC Reagent was added to the samples and resulting supernatant was processed using the PureLink Genomic DNA Kit (Invitrogen). DNA was eluted in DNA-Free PCR Water (MoBio). No contamination from either reagents or experimental procedures were observed.

### 454 sequencing and sequence analysis

16S rRNA V1-3 amplicon libraries were prepared from sample DNA using AccuPrime HF Taq (Invitrogen, 12346–086) and universal primers flanking variable regions V1 (27F, 5’-AGAGTTTGATCCTGGCTCAG-3’) and V3 (534R, 5’-ATTACCGCGGCTGCTGG-3’). For each sample, the universal primers were tagged with unique sequences (barcodes) to allow for multiplexing/demultiplexing [30]. The following PCR conditions were used; 2ul PCR buffer, 0.15ul AccuPrime Taq, 4pmol of each primer, 2ul sample DNA and DNA-Free PCR water to 20ul. Reactions were performed in 30 cycles of 95’C (20sec), 56’C (30sec), and 72’C (5min) thermal cycling. PCR products were purified with the Agencourt AMPure XP Kit (A63880) and quantitated with the Quant-iT dsDNA high-sensitivity assay kit (Invitrogen, Q33120). Approximately equivalent amounts of each PCR product were then pooled and purified with a Qiagen MinElute column (Qiagen, 28004) into 30 mL TE prior to sequencing at the NIH Intramural Sequencing Center. Amplicon libraries were sequenced on a 454 GS FLX (Roche) instrument using titanium chemistry.

### Sequence analysis pipeline

Sequences were processed using mothur version 1.31.0 [31] as previously described [16]. Briefly, 454 flowgram data were denoised, and primer and barcodes were trimmed using trim.flows and shhh.flows command (tolerant of primer errors ≤1, barcode errors ≤2, homopolymers ≤8 and length ≥200 bp). Sequences were then aligned through SILVA reference database [32]. Chimeras from PCR artifacts were identified and removed using mothur implemented UCHIME [33]. Next, sequences were classified to the genus level using a ribosomal database project naïve Bayesian classifier [34]. Operational taxonomic units (OTUs) were defined at 97% similarity using average neighbor joining.

Species-level classification for *Streptococcus* was performed using pplacer software package [16, 35]. Briefly, streptococci sequences were acquired via get.lineage command in mothur, and each sequence was then placed on a phylogenetic reference tree, which was generated with high-quality 16S rRNA reference sequences (obtained from RefSeq database and RDP type species), with pplacer software (parameter settings; --keep-at-most 1000 --max-pitches 1000, and likelihood cutoff 0.50).

### Validation of *S*. *salivarius* sequences

Since a previous report suggested *recA* gene comparison can differentiate *S*. *salivarius* from *S*. *vestibularis/S*. *thermophilus* [36], we designed a primer set specific to the *S*. *salivarius recA* gene (recA_F: 5’-TACGCAAATCAAAGGGACTGGTGAT-3’, recA_R: 5’-ACTAGCGATTTTTACAAGCTCACCT-3’). Specificity of primers was confirmed by PCR (Additional file 6a). In addition, to further validate *S*. *salivarius* speciation based on 16S rRNA gene sequences, we performed quantitative PCR of 9 selected samples (Additional file 6b). For quantitative PCR, relative *S*. *salivarius* levels were calculated by Ct values of *recA* gene, which were normalized by Ct value of pan-*Strepcoccus* primer (F: 5’- GTACAGTTGCTTCAGGACGTATC-3’, R: 5’- ACGTTCGATTTCATCACGTTG-3’), and for 16S sequencing data, *S*. *salivarius* levels were calculated with reads classified as *S*. *salivarius* divided by all *Strepcoccus* reads.

### Clinical metadata and parameters

Clinical metadata was collected, including subject’s gender and breastfeeding status. The clinical parameters of skin pH, TEWL and water capacitance were assessed at the left forearm of each infant as previously described [37].

### Statistics

Statistical analysis was performed with R software version 3.1 (Vienna, Austria) [38]. 1.5XIQR (Inter-quartile range) rule was used for defining outlier samples. Chao richness and Shannon diversity index were calculated based on OTU abundance. Chao richness, Shannon diversity and relative abundance of bacteria representing ≥1% abundance on the genus or species level were compared between both randomization groups at each sampling site with the Wilcoxon rank sum test with calculation of exact *P*-values. *P*-values were adjusted for multiple comparison by Benjamini Hochberg correction [39]. Similarity in bacterial community structure between the emollient and control groups was visualized by principal component analysis (PCoA) of matrices containing the Theta similarity index between each possible sample pair, and tested by AMOVA [40]. OTUs contributing to the dissimilarity between emollient and control samples were identified by Spearman correlation of the relative OTU abundances with the first PCoA axis. For analyzing contribution of selected taxa, reads mapped to designated taxa was removed and relative abundance was rescaled to 100%. Statistical significance for all tests was ascribed to a two-sided alpha level of the adjusted *P*-values <0.05.

## Results

We analyzed skin swabs from infants aged 6 months who had been randomized shortly after birth to use (n = 11) or not use (n = 12) once daily application of an emollient to the whole-body surface. Skin samples were obtained from six sites: both left and right cheeks, dorsal forearms, and volar forearms. All 23 infants had a family history of atopy. Clinical evaluation showed one infant from emollient group and three from control group developed AD over the course of the study period. To exclude the potential confounding effects of AD on skin bacterial communities [15, 16], we limited the bacterial diversity analyses to infants who did not develop AD (emollient group n = 10, control group n = 9), unless otherwise specified. We sequenced 114 samples (19 subjects, left and right of three skin sites for a total of six skin sites) with a total of 643,999 high-quality reads of the V1-3 region of the bacterial 16S rRNA gene (5,529 median reads/sample, > 3,000 reads/sample in 95.6% of samples, 275bp median read length). The numbers of reads/sample were evenly distributed between the emollient and control groups (S1 Fig). Sequencing provided sufficient coverage to analyze dominant members of skin bacterial communities in the emollient group and controls at all three sampling sites (S2 Fig). Of the subjects, ten infants were female (43.5%) and no twins were in this cohort. At the sampling timepoint, breastfeeding was reported in twenty infants (87%), of which nine were exclusively breastfed. Stratified analyses based on diet was not feasible due to the mixed diets of breastfeeding, formula, and solid foods in this cohort.

### Skin pH is lower in the emollient group than in the control group

**Fig 1:**
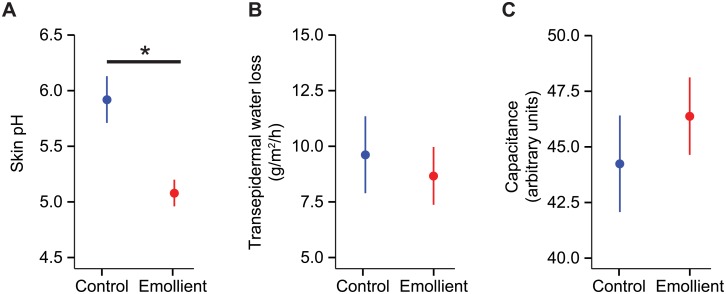
Skin assessments in infants who received emollients as compared to controls. (A) Skin pH, (B) transepidermal water loss and (C) water capacitance shown as mean ± standard error of the mean. *P < 0.05 (Wilcoxon rank sum test).

To identify possible alterations in clinical parameters correlating to the application of emollients, we measured the skin pH, transepidermal water loss (TEWL), and water capacitance in all infants. The skin pH was significantly lower in the emollient group than in controls (*P* = 0.02) (Fig 1A). TEWL was slightly lower and the skin water capacitance was slightly higher in the emollient versus the control groups. While lower TEWL and higher skin capacitance have been demonstrated to occur with emollient use in earlier timepoints after routine emollient use [41], results from this and the Schario *et al*. studies were not statistically significantly different with longer term emollient use (Fig 1B and 1C).

### The number of different bacterial species is higher in the emollient group than in controls

**Fig 2:**
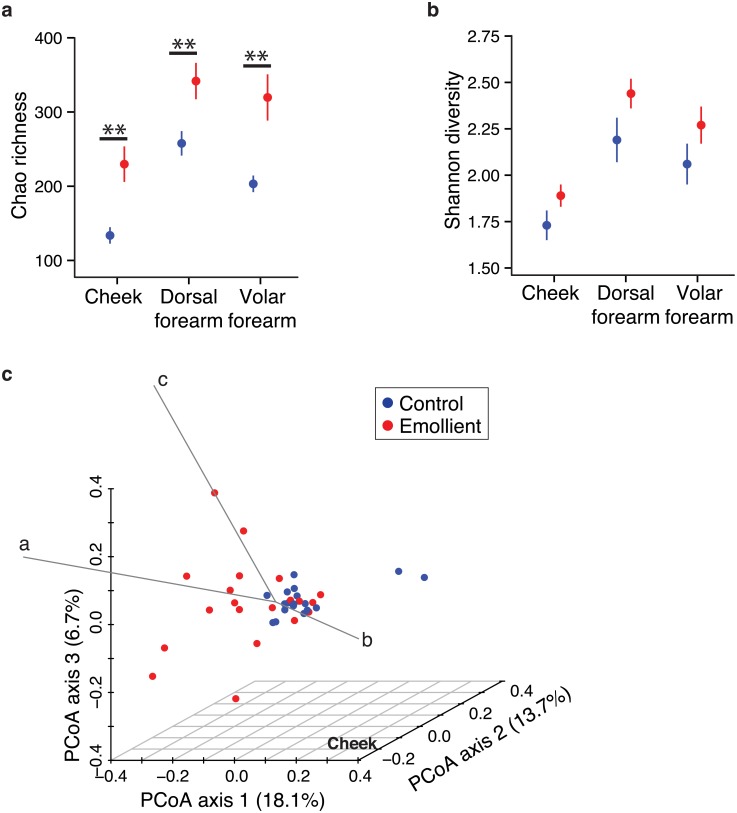
Diversity indices of skin bacterial communities. (A) Chao richness and (B) Shannon diversity shown as mean ± standard error of the mean. *P < 0.01 (Wilcoxon rank sum test). (C) The Yue-Clayton similarity index at the cheek, plotted in a principal coordinate analysis (PCoA). Samples from the right and the left body site are shown separately. Percentage variation attributed to PCoA axes are indicated at the axis labels. Biplot arrows indicate bacterial taxa significantly contributing to dissimilarity between samples, the lengths of arrows indicate the amount of contribution to dissimilarity along axis 1 (Spearman correlation). Letter codes for bacterial taxa, Spearman correlations with axes and associated *P*-values are shown in Table 1.

To examine if emollient therapy potentially altered the skin microbiome, we analyzed the structure of skin bacterial communities in samples from the emollient and control groups using the Chao richness and Shannon diversity measures. Chao richness, an ecological measure of the total number of different bacterial taxa (referred to as operational taxonomic units, OTUs) was significantly higher in the emollient group than in controls at all sampling sites (cheeks, *P* = 0.002; dorsal forearms, *P* = 0.009; volar forearms, *P* = 0.005) (Fig 2A). These results indicated that use of emollients was associated with the presence of more bacterial taxa than in controls. Given the skin site-specific differences of skin microbial communities [14], we next sought to investigate site-specific differences between emollient use and the number of bacterial taxa. We found that cheek samples in both the emollient and control groups harbored the fewest number of different bacterial taxa as compared to the dorsal forearms (emollient group, *P* = 0.002; control group *P* < 0.001) and volar forearms (emollient group, *P* = 0.03; control group *P* < 0.001) (Fig 2A). The dorsal forearm samples had more bacterial taxa than the volar forearms within the control group (*P* = 0.004). However, the number of bacterial taxa compared between the dorsal forearms and volar forearms were similar within the emollient group (*P* = 0.5), suggesting reduction of site-specific differences. In addition, since both microbial richness and clinical parameters (especially pH and capacitance) were affected by emollient usage, we analyzed the association between microbial richness and clinical parameters (S3 Fig). Although Chao richness failed to correlate with skin pH and capacitance, we observed moderate correlation between skin pH and relative abundance of a specific bacterial genus, *Haemophilus* spp, on the cheek and dorsal forearm, suggesting association of skin acidity with colonization of specific microbes (S3 Fig).

Shannon diversity is another ecological index that, in addition to richness, accounts for evenness or the relative proportion of bacterial taxa. Similar to Chao richness, Shannon diversity appeared greater in the emollient group than in controls at all sampling sites, but was not statistically significant (Fig 2B). In investigating possible site-specific differences between emollient use and Shannon diversity, we found that the cheeks in both the emollient and control groups had the lowest Shannon diversity as compared to the dorsal forearms (emollient group, *P* < 0.001; control group *P* = 0.002) or volar forearms (emollient group, *P* = 0.003; control group *P* = 0.02). Shannon diversity was greater at dorsal forearms than at volar forearms within the emollient group (*P* = 0.03), but not within the controls (*P* = 0.2). Thus, the cheeks generally harbored less diverse bacterial communities as compared to the two arm sites.

### *Streptococcus* drives dissimilarity between emollient samples and controls

We next analyzed the similarity between the bacterial communities in both the emollient and control groups. For all sample pairs, we calculated the Yue-Clayton similarity index (θ), an ecological measure of the similarity between two samples which considers the number of shared and distinct bacterial taxa present and their relative abundances in the two samples. θ = 1 indicates identical communities and θ = 0 indicates absolute dissimilarity of communities [42, 43]. We compared the similarity of skin bacterial communities between the emollient and the control groups by principal coordinate analysis (PCoA) and analysis of molecular variance (AMOVA) [40] of θ. In PCoA, a shorter distance between points indicated more similarity between the samples represented by these points. In AMOVA, a *P* < 0.05 indicated that the skin bacterial communities of the emollient and control groups were significantly different from each other [40]. The cheek skin bacterial communities of emollient samples clustered separately from those of control samples (AMOVA *P* < 0.001), indicating less similarity between groups (Fig 2C). At the dorsal and volar forearms, clustering of emollient and control samples was not observed (AMOVA for dorsal and volar forearms *P* = 0.16), indicating more similarity between groups (S4 Fig). Thus, only the cheek samples demonstrated a site-specific dissimilarity of skin bacterial communities between the emollient and control groups.

To determine which bacterial taxa contributed to the clustering (i.e., dissimilarity) between samples of the emollient and control groups, we correlated the relative abundance of bacterial taxa in samples of both groups with the PCoA. Interestingly, only three taxa consistently contributed to the clustering of emollient and control samples with a medium to large correlation coefficient at all three sampling sites: *S*. *mitis* group, *S*. *salivarius* and *Rothia* spp. (Table 1 and Fig 2C). In the cheek samples, shifts in relative abundances of *S*. *mitis* group and *S*. *salivarius* strongly correlated with differences between the emollient and the control groups. The opposing vectors in the PCoA representing these species indicated a higher proportion of *S*. *mitis* group (correlation to axis variation *r*_*s*_ = 0.68, *P* < 0.001) but a lower proportion of *S*. *salivarius* in the control group than the emollient group (*r*_*s*_ = -0.93, *P* < 0.001) (Table 1 and Fig 2C). Since the *S*. *salivarius* 16S rRNA gene sequence is highly similar to other species in the *S*. *salivarius* group, especially *S*. *thermophilus*, we further confirmed the existence/abundance of *S*. *salivarius* in our samples using *S*. *salivarius*-specific PCR (S5 Fig).

**Table 1:** Bacterial taxa that most significantly contribute to variation in principal coordinate analysis.

Sampling site	Taxonomy^a^	Spearman corr. with axis 1	*P*-value of correlation
**Cheek**	*Streptococcus salivarius* (a)	-0.93	<0.001
	*Streptococcus mitis* group (b)	0.68	<0.001
	*Rothia* (c)	-0.55	<0.001
	*Veillonella* (d)	-0.54	<0.001
	*Actinomyces* (e)	-0.47	0.002
	*Gemella* (f)	0.41	0.01
	*Corynebacterium* (g)	-0.41	0.01
	*Propionibacterium* (h)	-0.38	0.02
**Dorsal forearm**	*Propionibacterium* (a)	-0.83	<0.001
	*Streptococcus mitis* group (b)	0.72	<0.001
	*Staphylococcus* (c)	-0.59	<0.001
	*Granulicatella* (d)	0.5	0.001
	*Rothia* (e)	0.49	0.002
	*Corynebacterium* (f)	-0.48	0.002
	*Streptococcus salivarius* (g)	0.47	0.003
	*Prevotella* (h)	0.46	0.004
**Volar forearm**	*Streptococcus mitis* group (a)	0.76	<0.001
	*Gemella* (b)	0.75	<0.001
	*Actinomyces* (c)	0.66	<0.001
	*Rothia* (d)	0.58	<0.001
	*Porphyromonas* (e)	0.55	<0.001
	*Prevotella* (f)	0.55	<0.001
	*Veillonella* (g)	0.48	0.002
	*Streptococcus salivarius* (h)	0.46	0.004

^a^Letters in parentheses indicate labels for taxa in Fig 2

To analyze the contribution of particular taxa to the clustering of the emollient and control groups, we consecutively removed sequences specific for *S*. *mitis* group, *S*. *salivarius* and *Rothia* spp., and re-calculated PCoA and AMOVA. Removal of the *S*. *mitis* group or *S*. *salivarius* resulted in a loss of clustering between the emollient and the control groups at all sampling sites, as demonstrated by a considerable increase of AMOVA *P*-values after removal of these particular taxa (S1 Table). Removal of *Rothia* spp. did not affect clustering between samples of the emollient and control groups, as AMOVA *P*-values only slightly changed after removal of *Rothia* spp. (S1 Table). These findings demonstrated that shifts in proportion of *S*. *mitis* group and *S*. *salivarius* were the main contributors to the differences between the emollient and the control groups at all three sampling sites.

### Proportion of *Streptococcus salivarius* is higher in the emollient group than in controls

**Fig 3:**
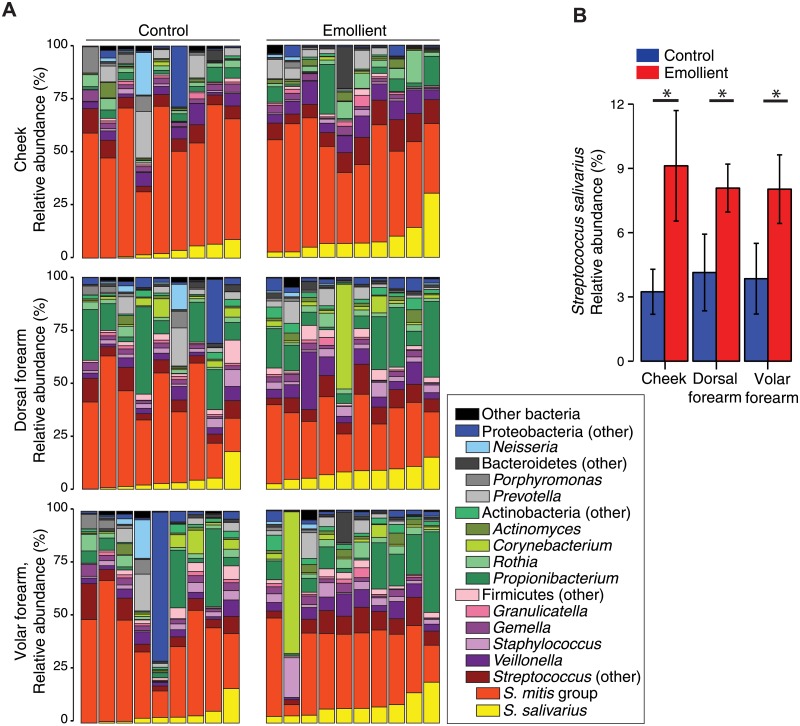
Bacterial taxonomic classifications in the skin of infants aged 6 months. (A) Relative abundance of order-genera that represented >1% of total 16S rRNA sequences with additional species level resolution for the two most abundant *Streptococcus spp*. Each bar represents the relative abundance of bacteria averaged from the right and left sides of each infant. (B) Relative abundance of *Streptococcus salivarius* in both infant groups separated by sampling site. The data are shown as the mean ± standard error of the mean. *P < 0.05 (Wilcoxon rank sum test).

Because *S*. *mitis* group and *S*. *salivarius* contributed to differences between samples from the emollient and control groups, we sought to investigate differences in the relative abundance of streptococcal species between these two groups. We observed that the genus *Streptococcus* predominated in both the emollient and the control groups at all three sampling sites. *S*. *mitis* group and *S*. *salivarius* were the most abundant species within this genus (Fig 3A). The proportion of *S*. *salivarius* in the emollient group was significantly higher than in controls at all sampling sites (Cheek, *P* = 0.02; dorsal forearm, *P* = 0.02; volar forearm, *P* = 0.02) (Fig 3B). In contrast, the proportion of *S*. *mitis* group appeared lower in the emollient group than in controls at all sampling sites, but this difference was not significant (S6 Fig). While *Rothia* spp. was also associated with clustering between samples of the emollient and control groups (see above), the proportion of this genus was not significantly different between the emollient and the control groups (S6 Fig). Moreover, in line with reduced skin pH in emollient group (Fig 1A), relative abundance of *S*. *salivarius* negatively correlated with pH (S6 Fig). These findings further suggested that *Streptococcus* spp. significantly contributed to the observed shifts in skin bacterial communities between emollient samples and controls.

### Proportions of *Streptococcus salivarius* and *Staphylococcus aureus* are inversely correlated with the severity of atopic dermatitis

**Fig 4:**
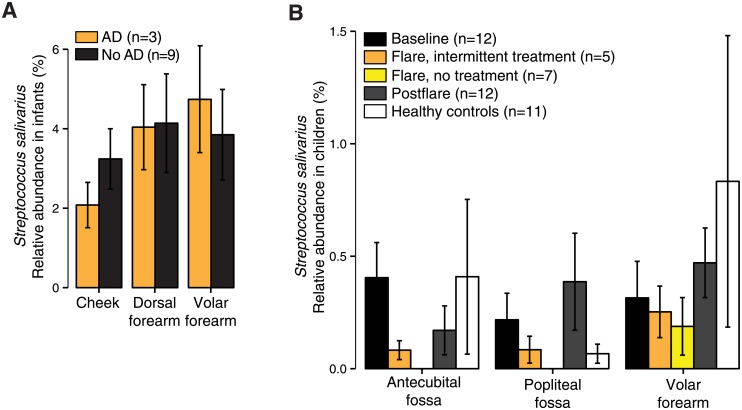
Relative abundance of *Streptococcus salivarius* in infants with atopic dermatitis (AD) versus without AD. (A) Relative abundance of *Streptococcus salivarius* in infants that did not receive emollients (A) Relative abundance of *S*. *salivarius* in children with AD (n = 12) at three disease states: baseline, flare, postflare; and in healthy control children. Data in (A) and (B) are shown as mean ± standard error of the mean. All comparisons are not statistically significant (P>0.05).

We next analyzed the proportions of *S*. *salivarius* in the healthy infants and the infants with AD in the control group. We found that the proportion of *S*. *salivarius* appeared lower in AD infants as compared to healthy infants at the cheeks (Fig 4A), which are sites of AD predilection in infants. The decreased *S*. *salivarius* proportion associated with AD was not statistically significant, presumably due to the low number of infants with AD (n = 3).

We therefore analyzed *S*. *salivarius* proportions in a previously published separate cohort of older healthy children and children with AD (2–15 years of age) [15]. In the prior study, AD patients were sampled during baseline disease state, disease flares, and post-flare (post-treatment) disease state, and *S*. *aureus* proportions were significantly increased during AD flares. Our analyses revealed decreased *S*. *salivarius* proportions during AD flares as compared to baseline and post-flare states (Fig 4B). This was notably observed in samples from the antecubital and popliteal fossae, which are sites of AD predilection in children. *S*. *salivarius* proportions were lower in non-treated AD flares as compared to intermittently treated AD flares, suggesting that use of skin-directed treatments (including emollients) in AD patients corresponds with higher proportions of skin *S*. *salivarius*.

## Discussion

AD is recognized as a complex multifactorial disease, including defective skin barrier and bacterial skin dysbiosis. Since emollients are considered an important therapeutic method to repair the impaired skin barrier in AD and are actively investigated as a method to prevent the development of AD [21], we sought to examine the effects of skin emollient use on the skin barrier and the skin bacterial communities by analyzing bacterial 16S rRNA gene sequences from skin swabs obtained from infants at-risk for developing AD.

The differences in the skin barrier parameters (pH, transepidermal water loss, and capacitance) between the treatment and control groups were consistent with previous findings of emollient use in skin [41]. Interestingly, skin pH was the only parameter statistically significantly different between the treatment groups in our cohort randomized to use 6 months of emollients and between the baseline and 16 week time points in the Schario *et al*. study using a treatment duration of 16 weeks. While Schario *et al*. noted statistically significant differences in transepidermal water loss and skin capacitance at earlier time points following initiation of emollient use, it remains unclear whether emollient associated changes in transepidermal water loss and capacitance diminish with chronic use of emollients or whether longer term use of emollients results in reduced treatment adherence [44]. While the persistent low skin pH would suggest that reduced frequency of emollient application is less likely to be contributing, potential mechanisms by which emollients may affect skin pH have been previously reviewed [45]. The acidic skin pH contributes to the barrier function of healthy skin, and a higher skin pH has been noted in atopic dermatitis as compared to healthy skin [46]. *In vitro* studies have demonstrated that higher skin pH results in greater adherence by *S*. *aureus* [47], suggesting altering skin pH is sufficient to modify skin bacterial communities. However, the low skin pH may contribute to the higher relative abundance of *S*. *salivarius* with emollient use and in healthier skin observed in the two patient cohorts analyzed.

Human microbiome studies have shown health-status differences in the bacterial diversity and number of bacterial taxa (richness). For example, skin samples from older AD children suffering disease exacerbations have decreased richness and diversity of skin bacterial communities, while treated AD patients and healthy age-matched controls have higher bacterial diversity [15]. Similar to the bacterial changes after skin-directed treatments in the older cohort, we found that emollient use correlated with an increased richness and a trend toward higher bacterial diversity as compared to no emollient use. Therefore, continuous application of the emollient used by the intervention group in this study was associated with more diverse skin bacterial communities in infants at risk for developing AD. Furthermore, the samples from the cheeks—a site of predilection of infantile AD [1, 5]—had less bacterial richness and diversity than the arm samples. In contrast, Capone and colleagues [48] studied healthy infants aged 4–6 months who had higher richness (Chao richness, 89 vs. 54) and diversity (Shannon index, 2.97 vs. 2.61) in forehead samples as compared to arm samples. While the differences between the studies may be attributed to the topographically distinct sites of the cheek and forehead, our cohort was selected for a family history of atopy. The lower bacterial diversity at a site of disease predilection in infants (cheeks) in our high-risk infant cohort is intriguing in light of the low gut microbial diversity correlated with a higher risk of AD identified by Ismail and colleagues [49]. Several other studies have observed that a diverse microbiome is more often associated with health [15, 44, 50]; however, the biological importance of maintaining a diverse bacterial community at different body sites requires further investigation.

Our analysis revealed that *Streptococcus* spp. significantly contributed to the observed differences in samples of the emollient and control groups. In particular, the proportion of *S*. *salivarius* was significantly higher in samples from the emollient group than in controls. *S*. *salivarius* colonizes human oral and nasopharyngeal epithelia a few hours after birth and persists there as a predominant commensal [51–53]. *S*. *salivarius* can also be found on the skin of healthy children and adults at various sites such as the volar forearm, antecubital fossa, and popliteal fossa, with a significant decrease in proportion from young childhood to adulthood (1.8–2.5% vs. <1%) [29]. The considerably higher proportion of *S*. *salivarius* in our infants aged 6 months (2.1–8.5%) is consistent with the higher proportion observed in oral cavity of children and may relate to exposure of the sampled skin sites to saliva. A lower relative proportion of *S*. *salivarius* positively correlated with presence of AD in infants and AD disease exacerbations in older children. This correlation was site-specific, particularly in age-specific sites of AD predilection: the cheeks in infants and the antecubital and popliteal fossae in older children. Our results raise additional questions regarding whether site-specific shifts in skin microbial communities in an at-risk population may influence the development of eczematous dermatitis [54] or whether the higher proportion of *S*. *salivarius* observed in healthy infants and less severe AD in older children may reflect a healthier state. In this study, the use of emollients may provide a favorable environment on the skin for an oral bacterium (e.g. *S*. *salivarius*) to persist.

*S*. *salivarius* strains have been shown to decrease production of proinflammatory cytokines and chemokines such as IL-1β, IL-6, IL-8, and TNF-α in *in vitro* epithelial assays [52, 55, 56]. Conversely, it can stimulate human peripheral blood mononuclear cells to produce high levels of IL-12 [57], which is involved in Th1 priming [58]. *S*. *salivarius* may be speculated to act as a driver towards a Th1 polarization and antagonist of the Th2 polarization in AD [10, 57, 59]. Hence, the presence of *S*. *salivarius* on the skin may dampen Th2-mediated skin inflammation. A limitation of the study was unavailability of skin biopsies to more extensively evaluate host immunity; however, inclusion of skin biopsies would have negatively affected study enrollment.

## Conclusions

The decreased skin pH and the increased bacterial richness and diversity associated with emollient use resembled the restoration of skin bacterial communities after skin-directed treatment of AD flares. *S*. *salivarius* had a statistically significantly higher relative abundance in the emollient versus the control group. *S*. *salivarius* proportion decreased with the development of AD in infants and of AD flares in children, whereas *S*. *aureus* showed the inverse development in children. The lower skin pH and immunomodulatory effects of *S*. *salivarius* may contribute to the therapeutic effects of emollient use in AD patients. Additional studies are needed to confirm pilot study results of emollients as a possible preventative therapy for AD and to investigate the mechanisms of how emollients affect the skin barrier and skin microbiota in an at-risk population.

## Supporting information

S1 Fig Histogram of the number of sequences per sample. Shown are high-quality reads of the V1-3 region of the bacterial 16S rRNA gene in a total of 114 samples from infants aged 6 months at risk for developing atopic dermatitis. Data from all sampling sites and both infant groups (emollients, n = 10; controls/no emollients, n = 9) where pooled to calculate the histogram.
(PDF)

S2 Fig Rarefaction analysis. This analysis was done for each group of infants aged 6 months (emollients, n = 10; controls/no emollients, n = 9) and each sampling site separately. Sampling time points represent the mean ± standard error of the mean of all infants in a group.
(PDF)

S3 Fig Association between clinical parameters and microbial community. Chao richness correlation with (A) pH and (B) capacitance. (C) Correlation of specific genera with pH of skin.
(PDF)

S4 Fig Principal co-ordinate analysis of the Yue-Clayton similarity index between all sample pairs at (A) the dorsal forearm and (B) the volar forearm. Samples from the right and the left body site of each infant are shown separately. Percentage variation attributed to PCoA axes are indicated at the axis labels. The biplot arrows indicate bacterial taxa significantly contributing to dissimilarity between samples at all sampling site. The lengths of arrows indicate the amount of contribution to dissimilarity along axis 1 as determined by Spearman correlation. Number codes for bacterial taxa, Spearman correlations with axes and associated *P*-values are shown in Table 1.
(PDF)

S5 Fig Confirmation of *S*. *salivarius* species using *recA* gene specific qPCR. (A) PCR result using pan-*Streptococcus* and *S*. *salivarius*-specific *recA* primers. sal(*S*. *salivarius*) and ther(*S*. *thermophilus*) gDNAs were used as template DNA. (B) Comparison of *recA* qPCR results and 16S sequencing data (see Method).
(PDF)

S6 Fig Relative abundance of (A) *Streptococcus mitis* group and (B) *Rothia* spp. in 6 months old infants. Infants either received emollients (n = 10) or served as controls/no emollients (n = 9). Data are shown as the mean ± standard error of the mean. (C) Correlation of *S*. *salivarius* with pH of skin (Spearman correlation).
(PDF)

S1 Table Removal of taxa from analysis of molecular variance (AMOVA). Each taxon in the cheek, volar or dorsal forearm samples was separately removed from AMOVA to assess changes in clustering of emollient and control groups.
(PDF)
