# Supplementary figures and images for: Emollient use alters skin barrier and microbes in infants at risk for developing atopic dermatitis
Source: PLoS One. 2018 Feb 28;13(2):e0192443. doi: 10.1371/journal.pone.0192443 (PMC5830298; doi:10.1371/journal.pone.0192443)

S1 Fig

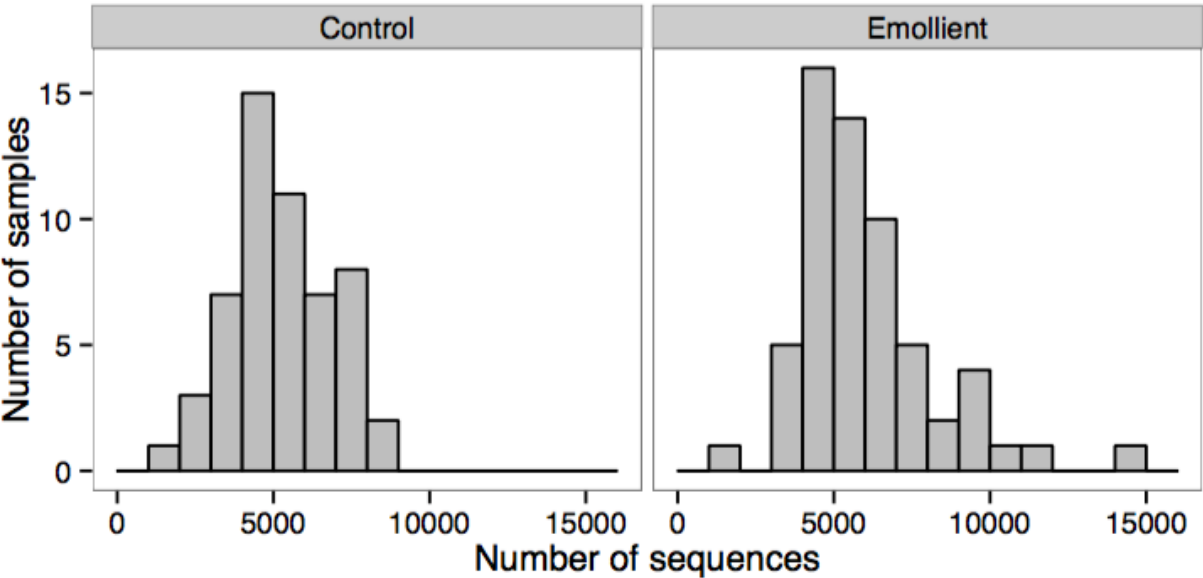

Supplement: S1 Fig — Shown are high-quality reads of the V1-3 region of the bacterial 16S rRNA gene in a total of 114 samples from infants aged 6 months at risk for developing atopic dermatitis. Data from all sampling sites and both infant groups (emollients, n = 10; controls/no emollients, n = 9) where pooled to calculate the histogram. (PDF) [file pone.0192443.s001.pdf]

S2 Fig

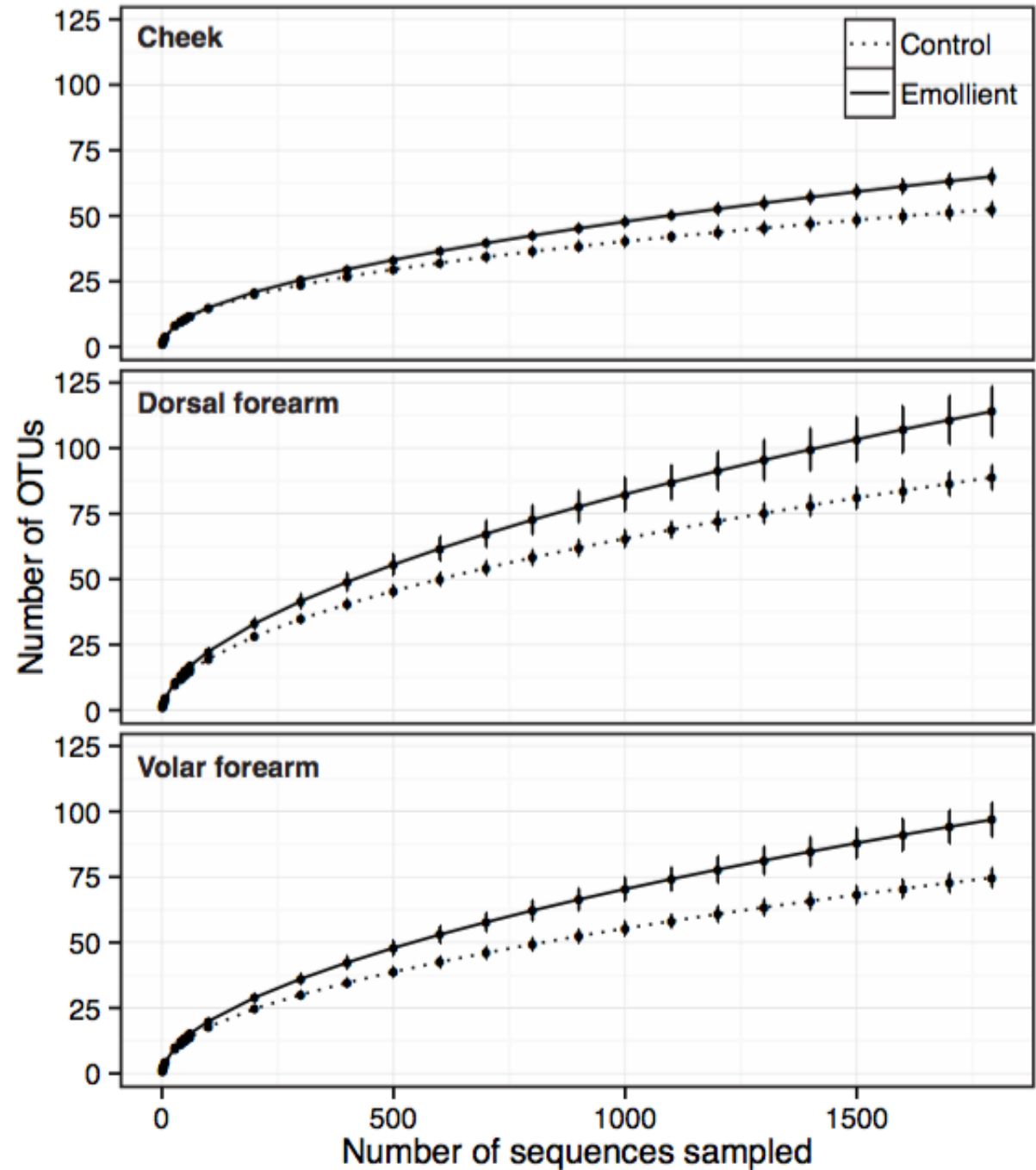

Supplement: S2 Fig — This analysis was done for each group of infants aged 6 months (emollients, n = 10; controls/no emollients, n = 9) and each sampling site separately. Sampling time points represent the mean ± standard error of the mean of all infants in a group. (PDF) [file pone.0192443.s002.pdf]

S3 Fig

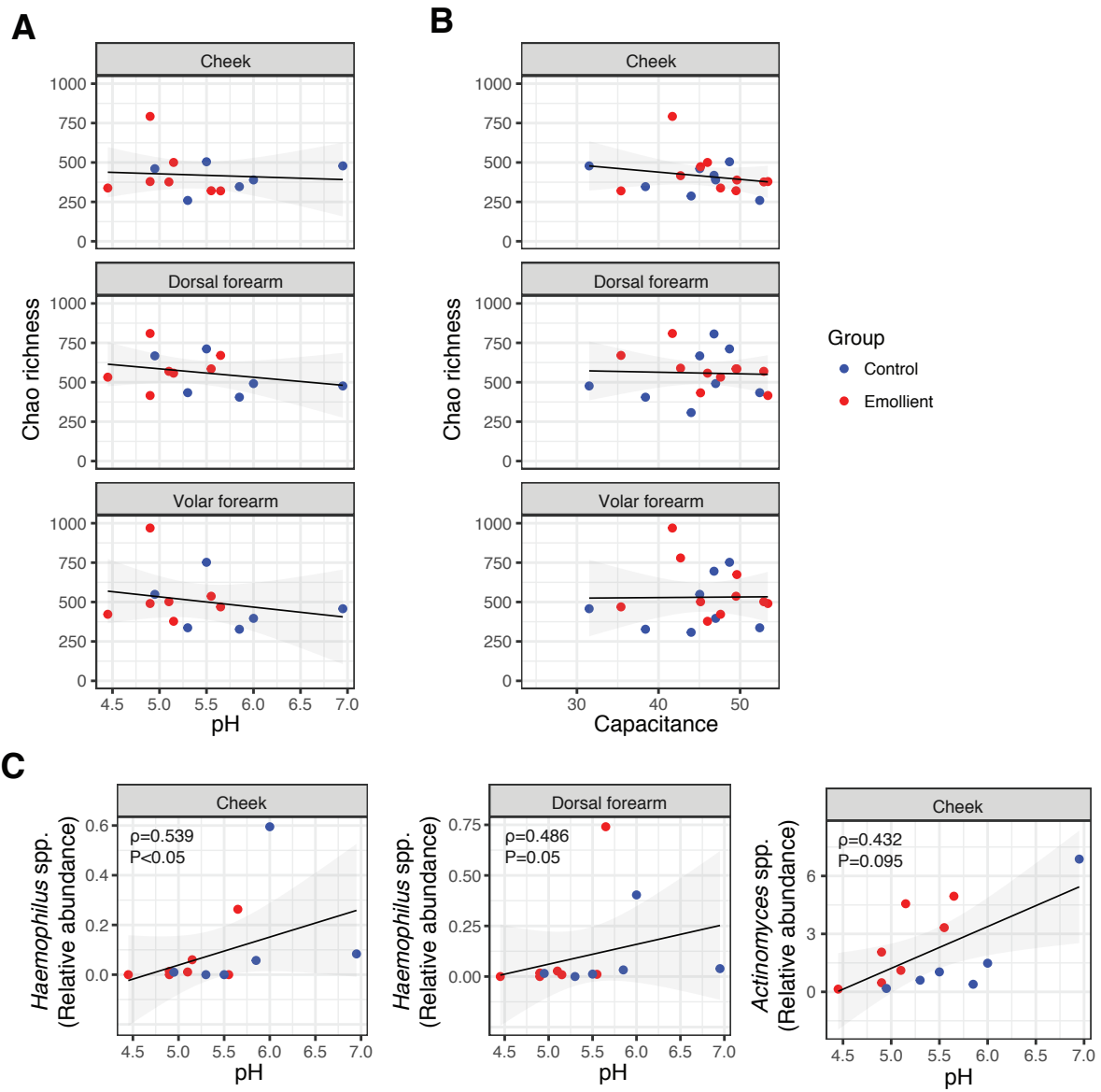

Supplement: S3 Fig — Chao richness correlation with (A) pH and (B) capacitance. (C) Correlation of specific genera with pH of skin. (PDF) [file pone.0192443.s003.pdf]

S4 Fig

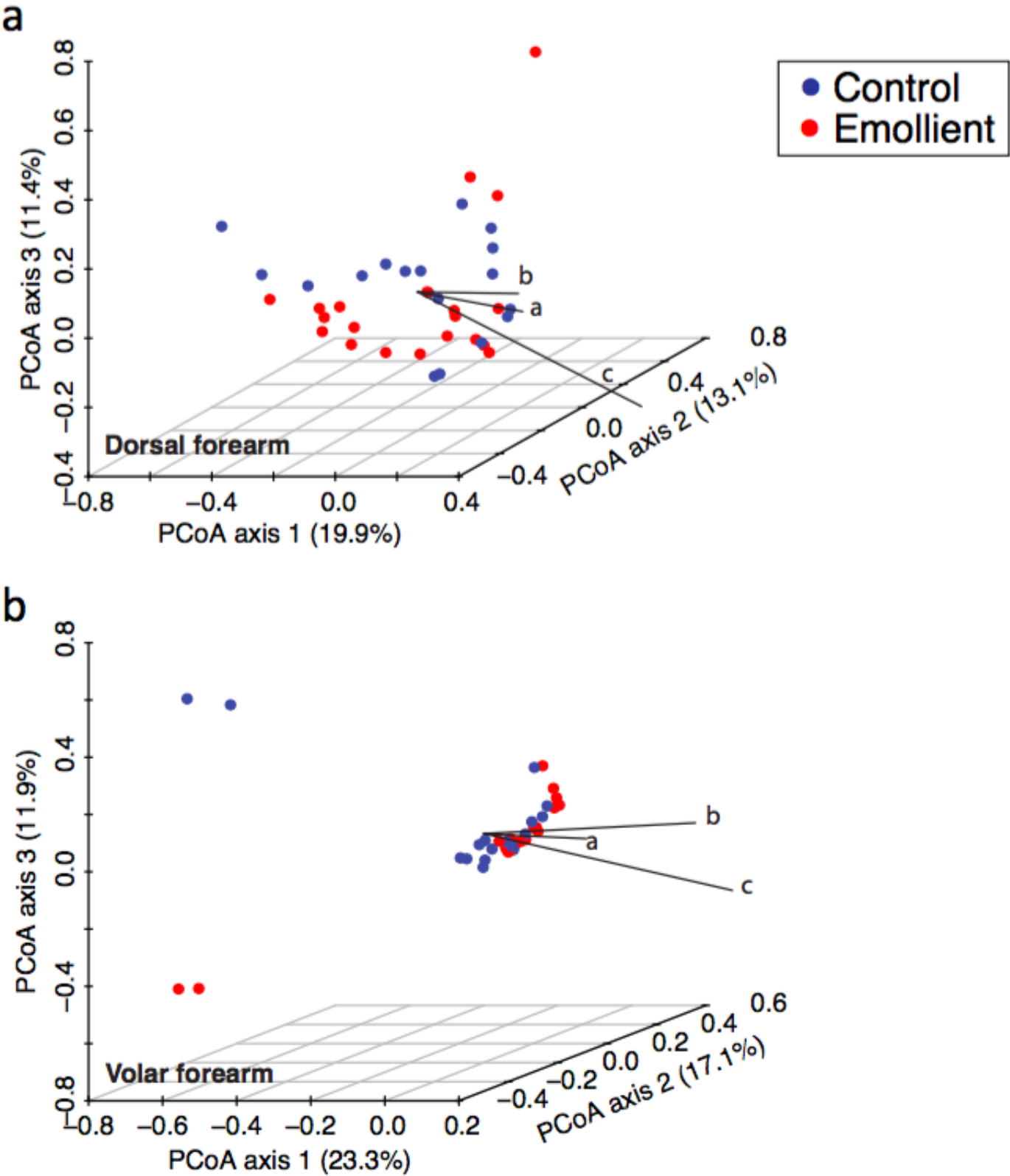

Supplement: S4 Fig — Samples from the right and the left body site of each infant are shown separately. Percentage variation attributed to PCoA axes are indicated at the axis labels. The biplot arrows indicate bacterial taxa significantly contributing to dissimilarity between samples at all sampling site. The lengths of arrows indicate the amount of contribution to dissimilarity along axis 1 as determined by Spearman correlation. Number codes for bacterial taxa, Spearman correlations with axes and associated P-values are shown in Table 1. (PDF) [file pone.0192443.s004.pdf]

S5 Fig

A

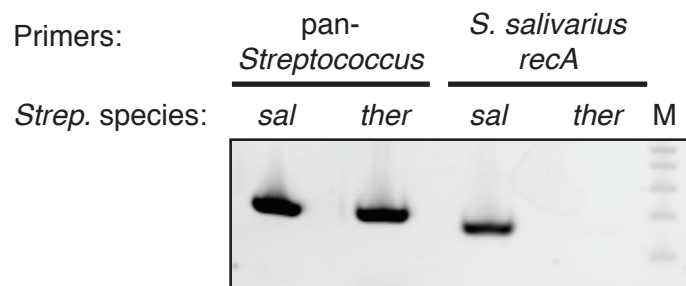

B

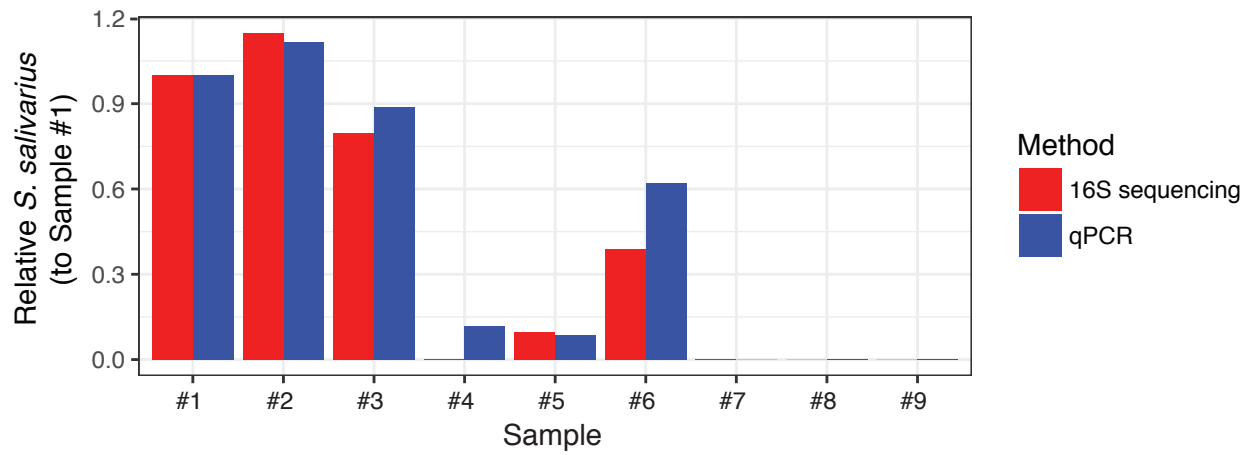

Supplement: S5 Fig — (A) PCR result using pan-Streptococcus and S. salivarius-specific recA primers. sal(S. salivarius) and ther(S. thermophilus) gDNAs were used as template DNA. (B) Comparison of recA qPCR results and 16S sequencing data (see Method). (PDF) [file pone.0192443.s005.pdf]

S6 Fig

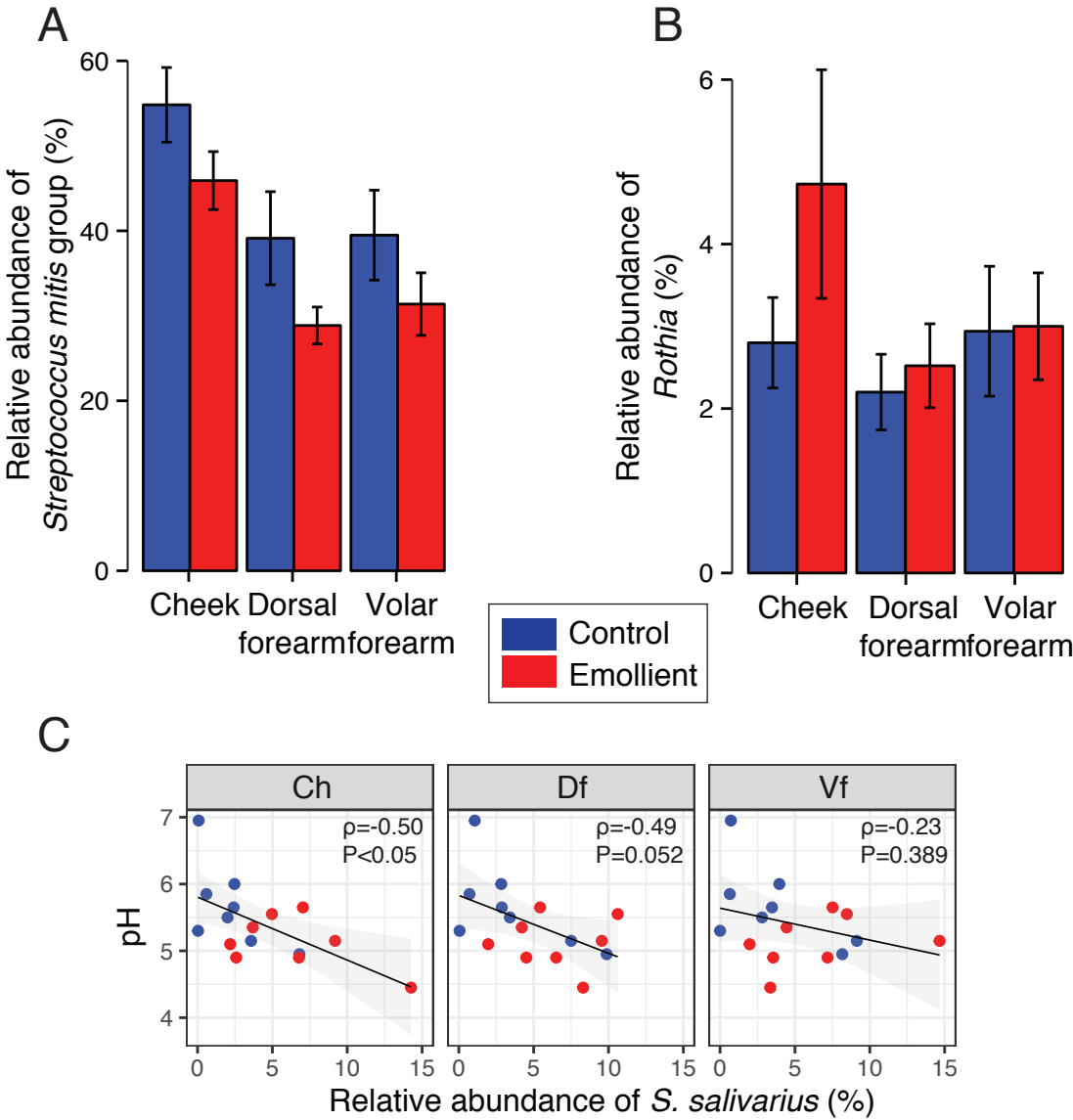

Supplement: S6 Fig — Infants either received emollients (n = 10) or served as controls/no emollients (n = 9). Data are shown as the mean ± standard error of the mean. (C) Correlation of S. salivarius with pH of skin (Spearman correlation). (PDF) [file pone.0192443.s006.pdf]
