# Supplementary material for: Emollient use alters skin barrier and microbes in infants at risk for developing atopic dermatitis
Source: PLoS One. 2018 Feb 28;13(2):e0192443. doi: 10.1371/journal.pone.0192443 (PMC5830298; doi:10.1371/journal.pone.0192443)
Supplement: S1 Table — Each taxon in the cheek, volar or dorsal forearm samples was separately removed from AMOVA to assess changes in clustering of emollient and control groups. (PDF) [file pone.0192443.s007.pdf]

S1 Table

| **Sampling site** | **Taxon removed from AMOVA[a]** | ***P*-value AMOVA** |
|---|---|---|
| **Cheek** | None | <0.001 |
| | *Streptococcus salivarius* (a) | 0.14 |
| | *Streptococcus mitis* group (b) | 0.09 |
| | *Rothia* (c) | <0.001 |
| | *Veillonella* (d) | <0.001 |
| | *Actinomyces* (e) | <0.001 |
| | *Gemella* (f) | <0.001 |
| | *Corynebacterium* (g) | <0.001 |
| | *Propionibacterium* (h) | 0.001 |
| **Dorsal forearm** | None | 0.16 |
| | *Propionibacterium* (a) | 0.08 |
| | *Streptococcus mitis* group (b) | 0.36 |
| | *Staphylococcus* (c) | 0.17 |
| | *Granulicatella* (d) | 0.16 |
| | *Rothia* (e) | 0.17 |
| | *Corynebacterium* (f) | 0.13 |
| | *Streptococcus salivarius* (g) | 0.47 |
| | *Prevotella* (h) | 0.14 |
| **Volar forearm** | None | 0.16 |
| | *Streptococcus mitis* group (a) | 0.36 |
| | *Gemella* (b) | 0.16 |
| | *Actinomyces* (c) | 0.17 |
| | *Rothia* (d) | 0.16 |
| | *Porphyromonas* (e) | 0.15 |
| | *Prevotella* (f) | 0.14 |
| | *Veillonella* (g) | 0.18 |
| | *Streptococcus salivarius* (h) | 0.33 |

[a]Letters in parentheses indicate labels for taxa in figure 2

AMOVA, analysis of molecular variance.
